# Morphohistological analysis of the prevalence and distribution of atheroma in the abdominal aorta and its branches: a cadaveric study

**DOI:** 10.1590/1677-5449.210014

**Published:** 2021-07-05

**Authors:** Naveen Kumar, Ashwini P. Aithal, Seemithr Verma

**Affiliations:** 1 Melaka Manipal Medical College (Manipal Campus), Manipal Academy of Higher Education, Manipal, Karnataka, India.; 2 Kasturba Medical College, Manipal Academy of Higher Education, Manipal, Karnataka, India

**Keywords:** abdominal aorta, iliac artery, atheroma, plaque, stenosis, aorta abdominal, artéria ilíaca, ateroma, placa, estenose

## Abstract

**Background:**

Aneurysms and atheromatous processes are prominent pathological features that are commonly associated with significant morbidity and mortality.

**Objectives:**

This cadaveric study was conducted to evaluate the morphometric and histological aspects of atheromatous plaque formation in abdominal aortas and their branches and their associated morphological variations, if present, characterized by loops, kinking, or tortuosity.

**Methods:**

The study was performed using 30 human cadavers (approx. 65-75 years). Frequency of occurrence of calcified plaques in the abdominal aorta and its branches and their morphometric measurements were noted and histological features were observed with the aid of Hematoxylin & Eosin staining.

**Results:**

Variations in the abdominal aorta and the common iliac artery were observed in 16.6% of specimens. Atheromatous plaque formation was seen in 2 specimens (1 specimen was associated with kinking) while in 3 other specimens only variation in normal structure (kinking/ tortuous artery) was observed. Histological analysis showed foamy macrophages and dense calcification, giving an atheromatous appearance.

**Conclusions:**

Cadaveric reports of the location, nature, and degree of plaque formation in the abdominal aorta and its branches are extremely important in clinical settings and for choosing treatment options.

## Resumo

**Contexto:**

Aneurismas e processos ateromatosos são características patológicas perceptíveis que costumam estar associadas a morbidade e mortalidade significativas.

**Objetivos:**

Este estudo em cadáveres teve como objetivo avaliar os aspectos morfométricos e histológicos da formação de placa ateromatosa na aorta abdominal e em seus ramos com suas subsequentes variações morfológicas caracterizadas por enrolamentos, acotovelamentos ou tortuosidades, se presentes.

**Métodos:**

O estudo foi realizado com 30 cadáveres humanos (aproximadamente 65-75 anos). Foi descrita a frequência de ocorrência de placas calcificadas na aorta abdominal e em seus ramos com as respectivas medidas morfométricas e características histológicas com auxílio da coloração por hematoxilina e eosina.

**Resultados:**

Variações na aorta abdominal e na artéria ilíaca comum foram observadas em 16,6% dos espécimes. A formação de placa ateromatosa foi observada em dois espécimes (um espécime foi associado a acotovelamento), enquanto em outros três espécimes houve apenas variação na estrutura normal (acotovelamento/artéria tortuosa). A análise histológica mostrou macrófagos espumosos e calcificação densa, o que gerou uma impressão ateromatosa.

**Conclusões:**

Os estudos em cadáveres sobre localização, natureza e grau de formação de placa na aorta abdominal e em seus ramos são extremamente importantes para os cenários clínicos e as opções de tratamento.

## INTRODUCTION

The abdominal aorta enters the abdomen passing through the aortic hiatus in the diaphragm at the level of the twelfth thoracic vertebra and ends at the fourth lumbar vertebra, dividing into right and left common iliac arteries. The abdominal aorta and its major branches supply all the organs in the abdominal cavity and the lower limbs.^1^ Striking variations in the origin and course of the principal branches of abdominal aorta have drawn attention from anatomists and surgeons for a long time. Accurate knowledge regarding its variations is of considerable practical importance during laparoscopy and various other surgical procedures.

Aneurysms and atheromatous processes are prominent pathological entities, commonly associated with significant morbidity and mortality, especially in recent decades. These entities constitute circumscribed dilations found in arteries, characterized by progressive focal dilatation of the vessel wall, involving all three layers: intima, media, and adventitia, and may progress to rupture or dissection.^2^ They are defined as aneurysm when the vessel diameter is greater than 3 cm or 1.5-fold its original diameter.^3^ An abdominal aortic aneurysm is a permanent localized dilatation of the abdominal aorta. Risk factors include male gender, age, prior vascular disease, hypertension, cigarette smoking, family history, and hypercholesterolemia.^4^ Atherosclerosis is the most common causative factor.^5^ Atherosclerotic lesions usually predominate in the branch ostia, bifurcations, and arterial bends, and they are directly related to arterial pressure and wall stress.^6^^-^8

From an anatomical standpoint, current knowledge and understanding of atherosclerosis supports the hypothesis that atheromatous plaque formation tends to occur more frequently near arterial bifurcations. Disruptions in normal laminar blood flow at an arterial division, or looped and tortuous nature of vessels, exert shear stress against the tunica intima, which is said to cause endothelial injury and inflammatory response.^9^^-^11 Despite this association, very few studies have been conducted to evaluate the existence of such a correlation between these two variables (i.e., looped nature of the vessels and presence of atheromatous plaque) and there is a lack of population-specific data. The paucity of the current literature necessitates further research utilizing more current specific histological techniques in order to establish a relationship between plaque formation and vessel tortuosity.

The present study was undertaken to evaluate the morphometric and histological aspects of atheromatous plaque formation in the abdominal aorta and its branches. Thus, this study aims to verify whether abnormal anatomy containing loops, bends, and tortuosity in the abdominal aorta and its branches and changing circulatory dynamics cause atheromatous plaques in adult human cadavers from the South Indian population.

## MATERIALS AND METHODS

Thirty formalin embalmed male cadavers of South Indian origin were procured from the Department of Anatomy. The sample size of 30 was based on a 95% confidence interval of 3.3%. The approximate age of the cadavers at death ranged from 65-75 years. No medical history was available on these cadavers. This study is in compliance with the Helsinki Declaration and with local ethical guidelines. The authors certify that they have obtained all appropriate consent forms and ethics committee clearance for the use of cadavers in this study. No patient data was used in the study. Cadavers with intra-abdominal pathology or any congenital anomalies, interfering with the dissection were excluded from the study.

The anterior abdominal wall was incised, and musculocutaneous flaps were reflected laterally. The peritoneal cavity was opened, abdominal organs were removed and the different branches of the abdominal aorta were identified. We then examined the frequency of occurrence of atherosclerotic or atheromatous plaques in the abdominal aorta and its branches. When plaques were found to be present, their vertebral level, exact location in the artery, and vertical and transverse diameters in centimeters were recorded using a scale. The severities of these plaques were also noted and rated by relative intimal area of involvement. The sizes of the plaques were divided into three groups, taking the size of the vessels into consideration. The plaques in the abdominal aorta were classified as mild (0–1 cm), medium (1–2 cm) and severe (> 2 cm), depending on their size.^12^ To evaluate the degree of atheromatous plaque formation, a small piece of the plaque was removed and preserved in 10% formalin. This was processed using the standard procedure, embedded in paraffin, and then thin sections were mounted on clean glass slides. These sections were stained with Hematoxylin and Eosin (H&E) stains and examined under the microscope using different magnifications for histopathological studies, and relevant photographs were taken. The photomicrographs of the plaque specimens were then classified based on a report by Stary et al.^13^

Stary et al.,^13^ for the American Heart Association’s Committee on Vascular Lesions, attempted to correlate the appearance of lesions noted in clinical imaging studies with histological lesion types in arterial wall and corresponding clinical syndromes. According to their report, Type I arterial lesions contain atherogenic lipoprotein, increased macrophages and formation of scattered macrophage foam cells. Type II lesions consist primarily of layers of macrophage foam cells and lipid-laden smooth muscle cells and include lesions grossly designated as fatty streaks. Type III is the intermediate stage between type II fatty streak and type IV atheroma, wherein the lesions contain scattered collections of extracellular lipid droplets and particles that disrupt the coherence of some intimal smooth muscle cells. Type IV lesions are characterized by a large, confluent, and more disruptive core of extracellular lipid. Lesions that usually have a lipid core may also contain thick layers of fibrous connective tissue and are classified as type V lesions and those with fissure, hematoma, and thrombus are type VI lesions.

## RESULTS

### Gross anatomy findings

**Figure 1:**
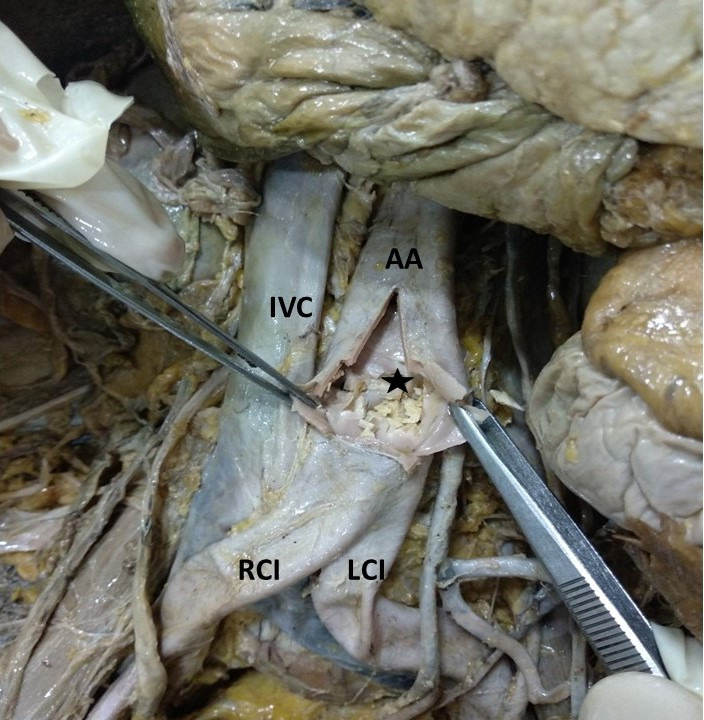
Dissection of the abdominal aorta (AA) showing the atheromatous plaque (★). RCI: right common iliac artery; LCI: left common iliac artery; IVC: inferior vena cava.

**Figure 2:**
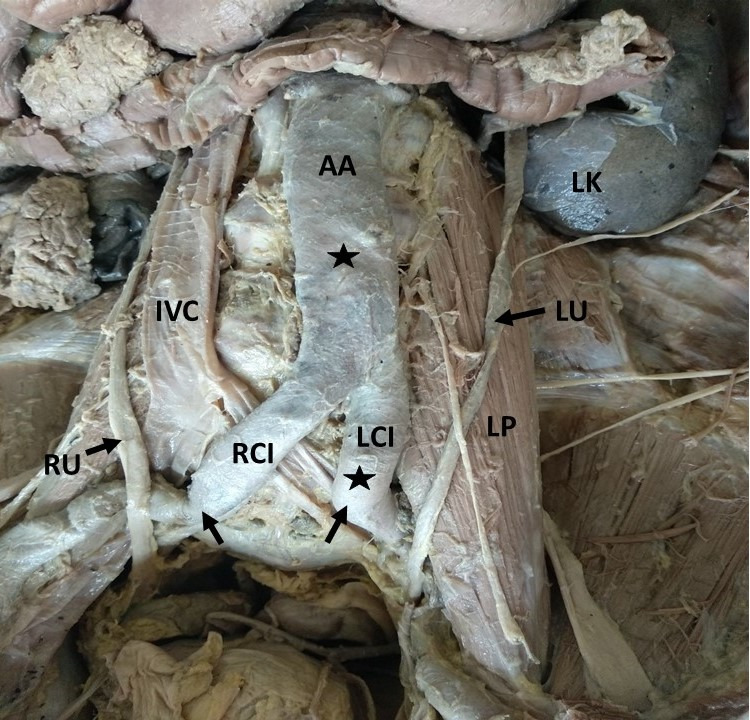
Dissection of the abdominal aorta (AA) showing the atheromatous plaque (★) in the abdominal aorta and left common iliac artery (LCI). Note the kinking (↑) in the right common iliac artery (RCI) and left common iliac artery (LCI); IVC: inferior vena cava; RU: right ureter; LU: left ureter; LK: left kidney; LP: left psoas major.

**Figure 3:**
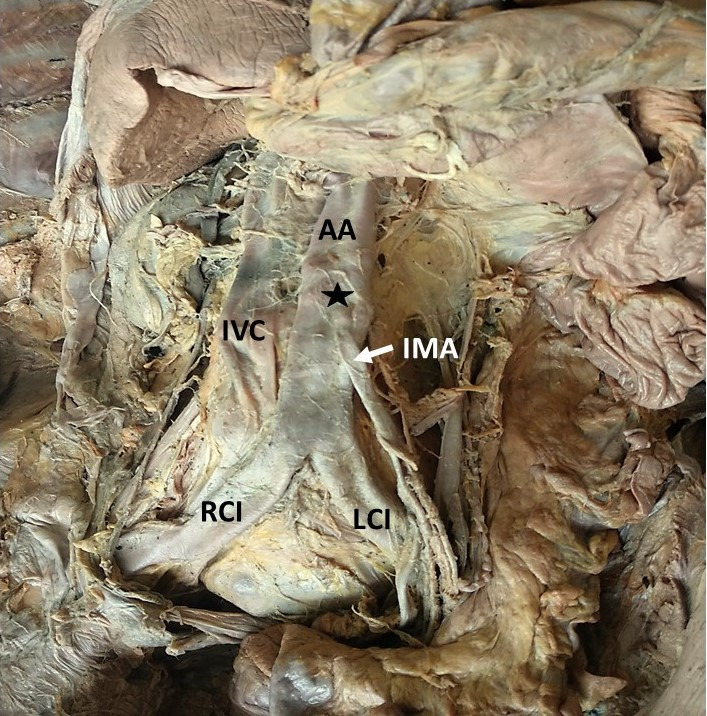
Dissection of the abdominal aorta (AA) showing the atheromatous plaque (★). RCI: right common iliac artery; LCI: left common iliac artery; IVC: inferior vena cava; IMA: inferior mesenteric artery.

**Figure 4:**
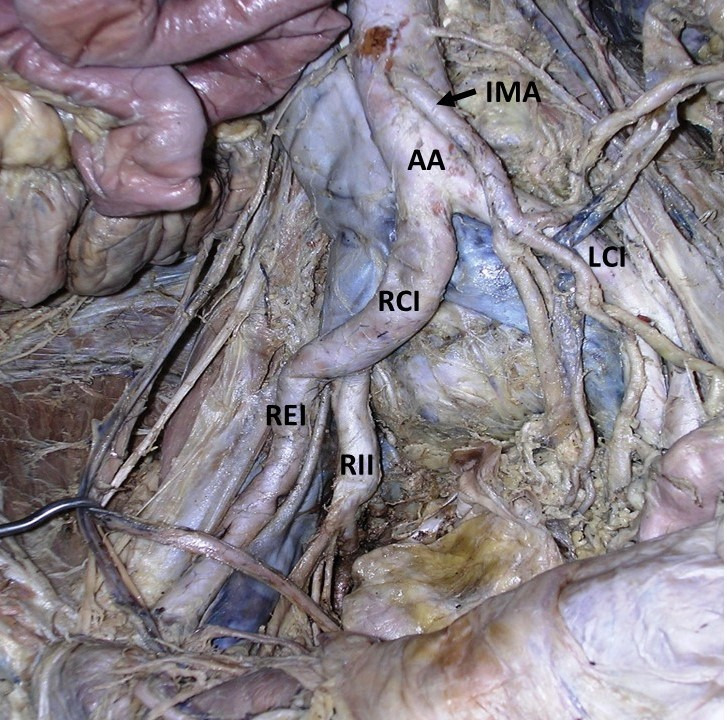
Dissection of the abdominal aorta (AA) and its branches showing the tortuous right common iliac artery (RCI). LCI: left common iliac artery; REI: right external iliac artery; RII: right internal iliac artery; IMA: inferior mesenteric artery.

Variations in the abdominal aorta and the common iliac artery were observed in 5 of the 30 specimens we studied (16.6%, with 95% CI of 3.3%). Atheromatous plaque formation was seen in 2 of these 5 specimens, (Figure 1), while in the other 3 specimens only variation in the normal structure was observed. In 1 of the 2 specimens with calcification, there was associated kinking of both right and left common iliac arteries (Figure 2). In this specimen, calcification was seen both in the abdominal aorta and common iliac arteries. In the other specimen, calcification was seen in the abdominal aorta, but kinking was not observed (Figure 3). Among the 3 specimens without calcification, we observed that one specimen had a tortuous right common iliac artery (Figure 4) while the other 2 specimens had kinking of the common iliac artery. The detailed morphometric measurements of these plaques, with their vertebral levels and distribution are shown in Table 1. All plaques were present on the anterior side of the vessel and were classified as mild or medium plaques by their size (< 2 cm).

**Table 1:** Showing the occurrence and distribution of atheromatous plaque and their detailed morphometric measurements.

Descriptions of plaques	Vertebral level	Morphometric measurements (cm)
1:		
Ø Plaque in the abdominal aorta	L3	Length: 3.5 cm
		Breadth: 1.6 cm
		
Ø Plaque in a left common iliac artery (associated with kinking)	Between L4 and L5	Length: 1.1 cm
		Breadth: 0.5 cm
2:		
Ø Plaque in the abdominal aorta	L2	Length: 0.5 cm
		Breadth: 0.2 cm

### Histopathological examination

**Figure 5:**
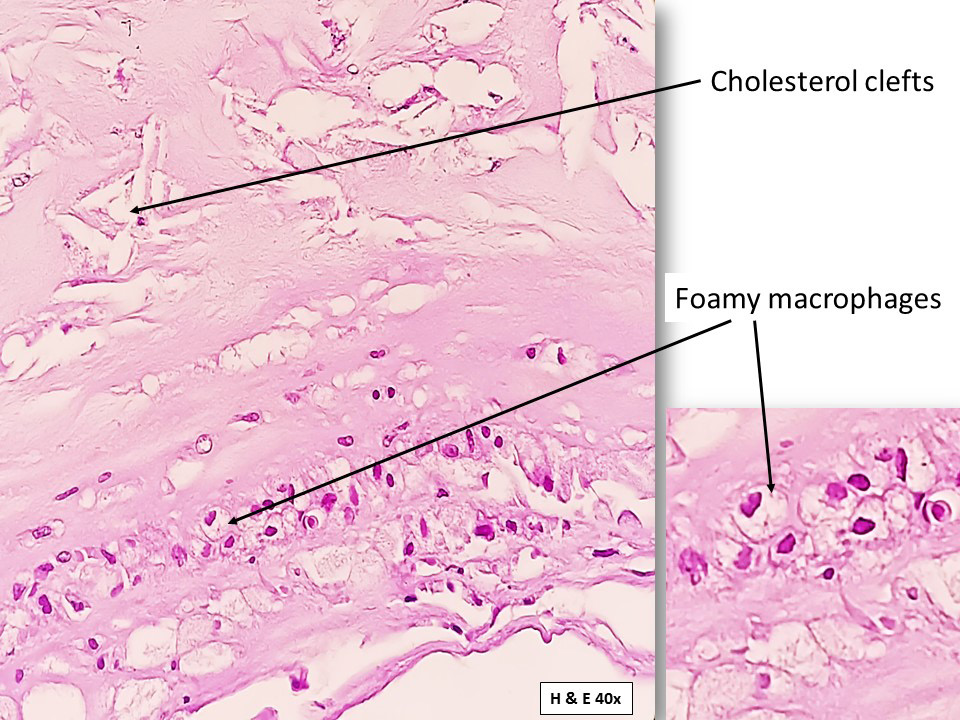
Photomicrograph showing the section of abdominal aorta stained using H&E stain. The section shows the features of atheromatous plaque formation in the vessel wall. (Magnification: 40X).

**Figure 6:**
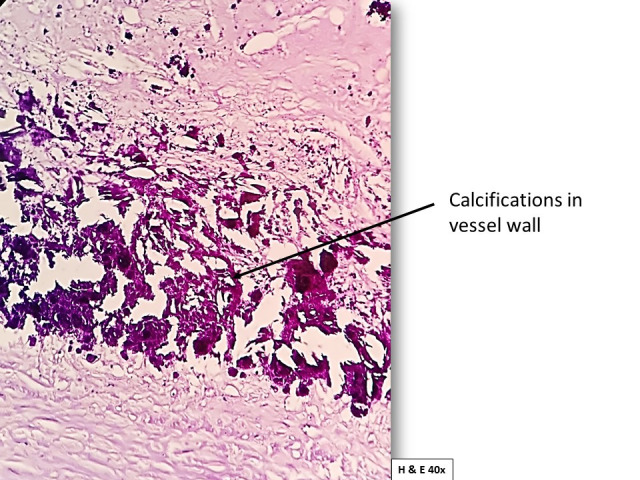
Photomicrograph showing a closer view of the vessel wall affected by the atheromatous plaque. (Magnification: 40X).

**Figure 7:**
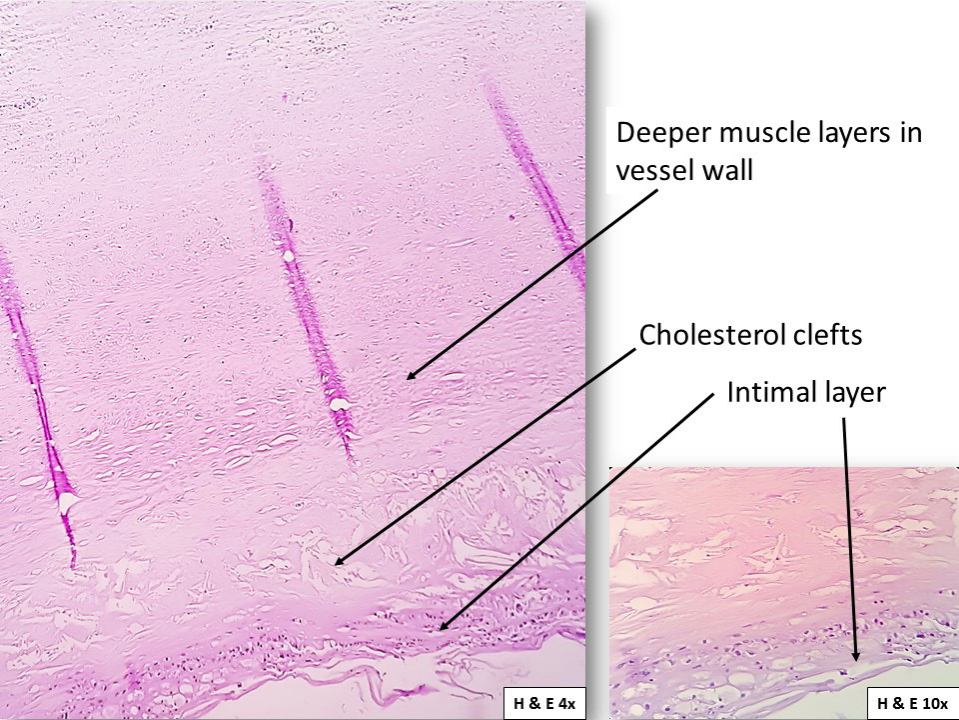
Photomicrograph showing the section of the aorta using H&E stain. The section shows the cholesterol clefts in the vessel wall. (Magnification: 4X and 10X).

The section of abdominal aorta studied with staining showed a fibrous cap composed of smooth muscles and foamy macrophages, overlying necrotic areas with cholesterol clefts, and dense calcification (Figures 5 and 6). Deeper layers showed smooth muscles with small to medium-sized blood vessels (Figure 7). Overall microscopic findings were suggestive of an atheromatous plaque. According to the Stary et al.^13^ classification, the plaque seen in the present study was a Type III lesion containing scattered collections of extracellular lipid droplets with foamy macrophages between the smooth muscle cells and was therefore classified as atheroma.

## DISCUSSION

An atheroma, or atheromatous plaque, is an abnormal accumulation of material (mostly consisting of calcium, debris, lipids, or fibrous connective tissue) in the inner layer of the wall of an artery. Numerous studies reported in the literature describe different types of aneurysms and atherosclerotic plaque formation in the thoracic or abdominal aorta. However, most of these studies are radiological studies. Studies of the internal dimensions and histopathology of the different types of atheromatous plaques in human cadavers have not yet been reported or described. Therefore, the present work was undertaken to study the prevalence and distribution of atheroma and associated structural changes in the abdominal aorta and its branches.

Kinking of the arteries is mainly seen in the carotids and aorta, may be congenital or acquired, and is most commonly seen in elderly patients suffering from atherosclerosis and hypertension.^14^ Presence of anatomical variants such as loops or kinking in the arteries alters the circulatory dynamics of blood flow, causing reduced velocity and drag stress, leading to mural changes in vessels. This phenomenon has been proved by many authors.^15^ In a study conducted by Boonruangsri et al.,^16^ the prevalence of tortuosity of common and external iliac arteries was 20%. No tortuosity of the internal iliac artery was observed in that study. In our study, kinking was observed only in common iliac arteries and was associated with atheromatous plaque formation. A similar study conducted by Purnendu et al.^17^ reported kinking, aneurysm, and tortuosity of common iliac arteries in two cadavers.

Literature highlights ageing as a determinant factor in morphological and functional changes in arterial walls, facilitating atherogenesis.^18^ In the elderly, the abdominal aorta frequently becomes elastic and tortuous, changing the angle and position of bifurcation. Aortic aneurysm is an important disease in the practice of vascular surgeons, with a prevalence of 2% to 4% in the general population and a male: female ratio of 5:1.^19^ It has been demonstrated that atherosclerosis is the main cause of aneurysms in adults. Other causes include connective tissue disorders, trauma, vasculitis, congenital, mycotic, and idiopathic.^20^ The prevalence of aneurysms, tortuosity, and kinking of the abdominal aorta and iliac arteries make them important primary considerations in operative planning. Recognition of these phenomena is important for optimizing radiation doses and in endovascular stent grafting or traditional surgery.^16^

Previous studies opine that, compared with the thoracic aorta, plaques in the abdominal aorta are usually more abundant, less discrete, more complicated, and more calcific. These differences may be related to local differences in aortic flow conditions and mechanical stress as well as to differences in the aortic wall structure, composition, and nutrition.^21^ They also found that plaque deposits, wall thinning, and aortic enlargement are maximal at the midpoint of the abdominal aorta and its bifurcation. This trend was also observed in the present study, as the atheromatous plaques were more commonly found in the distal part of the abdominal aorta, which is comparable with the literature.^12^

Atheromatous plaque formation causing narrowing of the vessels can lead to hypertension. Small pieces of plaque can separate, causing thromboembolic symptoms, resulting in ischemia in structures supplied by the arteries. Turbulent inflow at bends/bifurcations can damage the internal elastic fibers of the arteries, causing dissecting hematoma.^15^ Atherosclerosis or atheromatous plaque in the abdominal aorta are the main cause of stenosis of superior and inferior mesenteric arteries and stenosis of these branches may lead to acute or chronic mesenteric ischemia.^12^ Because of the deep location of the abdominal aorta in the retroperitoneum, a number of other vascular diseases like aortitis, congenital defects, and, most importantly, aneurysms are not easily detected on routine clinical and ultrasonographic examination.^22^ Therefore, there is an implicit need for accurate in-vivo measurements in all patients undergoing an endovascular repair in this vascular region.^22^ Thus, knowledge regarding the location and nature of plaques and degree of plaque formation in the abdominal aorta and its branches is extremely important for predicting clinical results and choosing the appropriate treatment (conservative, medical, or surgical) and for preventing progression of the disease and clinical syndromes such as mesenteric artery stenosis, renal artery stenosis, and splenic infarction.

## CONCLUSION

In the current study, variations in the abdominal aorta and the common iliac artery were observed in 16.6% specimens. Atheromatous plaque formation was seen in 2 specimens (in 1 specimen it was associated with kinking in common iliac arteries) while only variation in normal structure (kinking/ tortuous artery) was observed in the other 3 specimens. Histological analysis of the plaques showed foamy macrophages and dense calcification giving it an atheromatous appearance. These cadaveric reports deserve attention given their potential clinical application.
